# NRA-Net—Neg-Region Attention Network for Salient Object Detection with Gaze Tracking

**DOI:** 10.3390/s21051753

**Published:** 2021-03-04

**Authors:** Hoijun Kim, Soonchul Kwon, Seunghyun Lee

**Affiliations:** 1 Department of Plasma Bio Display, Kwangwoon University, 20 Kwangwoon-ro, Nowon-gu, Seoul 01897, Korea; hoi97@kw.ac.kr; 2 Department of Smart Convergence, Kwangwoon University, 20 Kwangwoon-ro, Nowon-gu, Seoul 01897, Korea; 3 Ingenium College of Liberal Arts, Kwangwoon University, 20 Kwangwoon-ro, Nowon-gu, Seoul 01897, Korea; shlee@kw.ac.kr

**Keywords:** autoencoder, convolutional neural network, deep learning, gaze tracking, image processing, salient object detection

## Abstract

In this paper, we propose a detection method for salient objects whose eyes are focused on gaze tracking; this method does not require a device in a single image. A network was constructed using Neg-Region Attention (NRA), which predicts objects with a concentrated line of sight using deep learning techniques. The existing deep learning-based method has an autoencoder structure, which causes feature loss during the encoding process of compressing and extracting features from the image and the decoding process of expanding and restoring. As a result, a feature loss occurs in the area of the object from the detection results, or another area is detected as an object. The proposed method, that is, NRA, can be used for reducing feature loss and emphasizing object areas with encoders. After separating positive and negative regions using the exponential linear unit activation function, converted attention was performed for each region. The attention method provided without using the backbone network emphasized the object area and suppressed the background area. In the experimental results, the proposed method showed higher detection results than the conventional methods.

## 1. Introduction

Video processing technology using deep learning has been studied in various fields, such as monochrome image colorization, object detection and recognition, super-resolution technology, character detection and object recognition. Particularly, in the field of object detection and recognition, the underlying method is salient object detection (SOD). The purpose of this method is to detect objects that are of interest to a person, detect objects that cause the line of sight when a person first sees a video or a single image, and track the line of sight based on the results. SOD predicts where the gaze is focused when gaze tracking without equipment. Dramatic scene changes when predicting gaze can cause tracking to fail. SOD can supplement gaze tracking information by learning the area where the gaze is concentrated and predicting the input image. SOD’s training dataset generates an area where the eyes of 20 to 30 people are focused through gaze tracking when displaying images on the screen instantly. This method recognizes objects and is used in various areas, such as scene classification, tracking and detection. Other typical object detection methods include the contour-based, division-based, and deep learning-based methods.

SOD is the creation of a saliency map by detecting objects that are of interest to people or objects that are considered to be the most important in a video or image. The correct saliency map of the SOD input image is called the ground truth. Typical split-based SOD methods include superpixel, contour-based, and deep learning-based methods.

The deep learning-based detection method does not require complicated pretreatments and posttreatment processes and shows a high detection rate. Most of the existing SOD methods have autoencoder structures, and the use of some methods results in a significant deformation of the model structure and loss function. The model structure can be improved to reduce losses during the feature extraction process due to the shallow structure and to maximize the error of the loss function during the learning process. Research is underway to improve the detection rate of salient objects based on deep learning-based methods. However, the detection rate drops due to the high degree of similarity between the object and the background or the existence of several objects.

The existing deep learning-based method typically includes a fully convolutional network (FCN) [1], which uses skip connection to minimize losses during the feature extraction process. However, various values are extracted at the feature extraction stage, and feature values can be expressed as negative in this process. Traditional methods focus on positive region values that do not utilize negative region values, which causes feature loss. Also, since deep learning research is being conducted based on the backbone network, a backbone network is always required. A backbone network is a feature extractor that has learned a lot of data in advance. Although it has the advantage of being able to select various features, the type of backbone is limited and shows poor performance when extracting features that have not been learned.

In this study, the negative region that was not used in the existing method is utilized. A new attention module is created by using the spatial attention technique in the negative region. This module minimizes loss of functionality during feature extraction. We also propose a deep learning model called the Neg-Region Attention (NRA), which aims to minimize the feature loss of salient objects due to complex environmental problems.The proposed method did not use a backbone network to extract desired features and does not require additional pre-trained weights. It aims to construct a relatively light model without a backbone network. In addition, it aims to improve the performance of feature extraction by providing a new module using the negative region.

## 2. Related Works

Deep learning is a machine learning algorithm that summarizes the core contents and features of complex data, with nonlinear transformation methods composed of multiple layers. In the existing machine learning algorithm, a person directly analyzes and evaluates by extracting the kind of features present in the data to be learned. However, in deep learning, necessary features are extracted and learned from the data that the machine automatically learns. In these studies, deep learning was developed as a convolutional neural network-based method with high data recognition and detection performance. In addition, deep learning technology has been established, and excellent performance methods have been developed in the field of SOD, where FCN is a typical example.

### 2.1. Hand Crafted-Based Detection Method

Superpixel-based split methods [2,3] split the salient object and background with the internal information of the image, such as brightness, color, contrast and texture. Because salient objects have movements in the video, the method considers the position of the object according to the time using a superpixel partition. Contour-based detection methods [4] include detecting salient objects using a fast Fourier transform and a Gaussian filter. Such a method maintains the contour of the object and shows a high detection rate, but it requires a large amount of calculation due to pre-processing and post-processing process requirements. In addition, the detection rate of salient objects decreases due to problems such as complicated backgrounds, high similarity between backgrounds and objects, or the existence of several objects.

### 2.2. Deep Learning-Based Detection Method

The deep learning-based SOD method shows high accuracy in trained images without requiring complicated pre-processing and post-processing processes. In the present research, this method is performed with an autoencoder structure, and it can be further classified to methods transforming the network structure and transforming the loss function. When the network structure is deformed, the loss and shallow structure in the process of extracting the features are improved, which consequently improves the results. When the loss function is transformed, the loss function is improved, which consequently minimizes the error in the learning process. Both methods show improved performance, and even when transforming the loss function, it is necessary to improve the network structure in the process of minimizing the error [5,6,7,8,9].

Recently, many deep learning-based detection methods have been studied to improve the performance of SOD. However, the detection rate drops due to problems, such as high similarity between the background and object and complicated background. In addition, feature loss occurs during feature extraction through several convolution layers.

### 2.3. Autoencoder

An autoencoder [10] is a type of artificial neural network used to compress and restore image data. It is a learning model with a structure similar to that of feed-forward neural networks (FNNs) [11]. Different from FNNs, the sizes of the input and output layers of an autoencoder are always the same.

**Figure 1:**
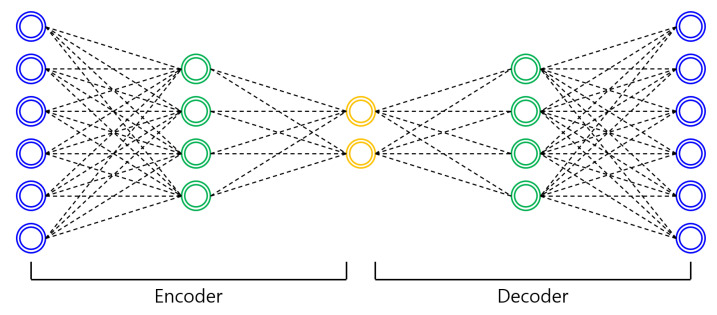
Autoencoder architecture example.

An autoencoder is largely composed of an encoder and a decoder. The encoder is likely a network that extracts features from the input data or compresses it into an internal representation. The decoder is a generation network that converts extracted features and compressed internal representations into the output. An autoencoder is a deep learning network structure that is often used in the field of SOD and partitioning. The autoencoder copies the input to the output only on the same side as the input layer with the same number of nodes in the hidden layer. Therefore, the number of nodes in the hidden layer is smaller than that in the input layer and the data are compressed, as shown in Figure 1. In this method, control is used to represent data efficiently. The upsampling of the decoding process causes feature loss as it is simply used in the process of increasing the size of the feature map.

## 3. Proposed Method

The proposed method, that is, NRA, has an autoencoder structure and research has been conducted to reduce losses that occur in the process of compressing and decompressing features and losses that occur in the process of expanding extracted features. Existing methods use the rectified linear unit (ReLU) [12] as an activation function in the encoder process of extracting features. ReLU treats negative regions as 0, so feature loss occurs, but the learning speed is fast. Instead of using ReLU, the conventional method uses a model in the backbone network where a large dataset is trained to prevent feature loss. The backbone network requires pre-trained weights, and there is a limit to model transformation. Most of the input images are also fixed and the model becomes heavier.

**Figure 2:**
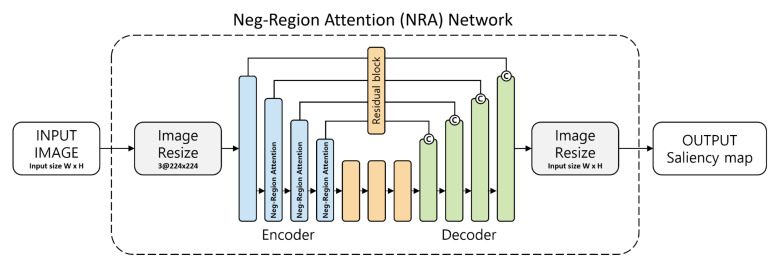
The overall flowchart of our Salient Object detection method. (Neg-Region Attention Network).

The proposed method aims to improve the heaviness of the model, change the size of the input image according to the user’s computer performance, and minimize the feature loss at the encoder stage. ELU is used as an activation function to avoid the feature loss problem that occurs in ReLU. The exponential linear unit (ELU) activation function is used in the encoding process to compress and extract features and utilize them in the negative region. The NRA provided for the extracted features can be used to suppress non-object areas in the negative areas and emphasize the contour and texture information of the objects in the positive areas. The decoding process, which enlarges the extracted features to the size of the input image, utilizes the features in the encoding stage through concatenation. Through this process, an improved saliency map is generated. The detection flowchart using NRA proposed in this paper is shown in Figure 2.

### 3.1. Feature Extraction Using the Proposed Attention

**Figure 3:**
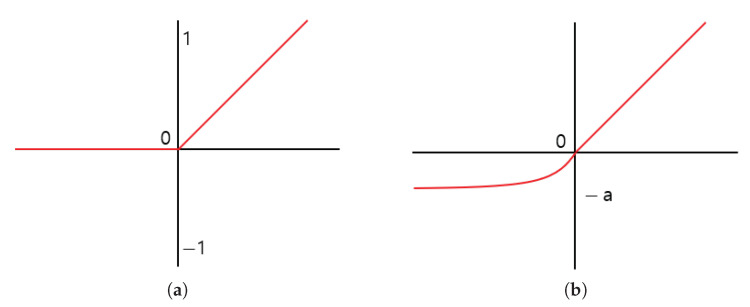
The rectified linear unit (ReLU) activation function graph before (**a**) and after (**b**) is an Exponential Linear Unit (ELU) activation function graph.

In a deep learning-based method, the convolution operation results involve negative and positive regions. The conventional method uses ReLU activation function to determine which node to pass to the next layer. The ReLU activation function treats the negative region as 0 and causes feature loss in the feature extraction process. Accordingly, the proposed method utilizes the negative region and uses the Exponential Linear Unit (ELU) [13] activation function to prevent feature loss. The positive region of the ELU activation function is processed similarly as the ReLU activation function, and the negative region has a convergence form of (1) and (2). Equations (1) and (2) are the equations of the ReLU and ELU activation functions, respectively, where the exp() function was used in the negative region in ELU. The graphs of the activation functions are presented in Figure 3.

(1) ReLU(x)=max(0,x)

(2) $$ELU\left(x\right)=\left\{\begin{array}{cc} x, & \mathrm{if}\;x\geq 0\\ a(e^{x}-1), & \mathrm{if}\;x<0. \end{array}\right.$$

As shown in Figure 3, the graph of the ELU activation function directly outputs the input in the positive region, but in the negative region, it is normalized so that it is not outputted immediately and converges to −a. By setting the values of a, the influence of the negative region can be limited. The proposed method uses all the characteristics of the positive and negative regions by setting a to 1, but the negative region has little effect.

When the value of *a* is set large, the effect of the negative region becomes large in the complete data representation, and the texture information around the boundary information in which the amount of data change is large is expressed in various ways. The proposed method uses integrated texture information rather than various texture information in the negative area and sets the value of a to 1 to suppress non-object areas.

**Figure 4:**
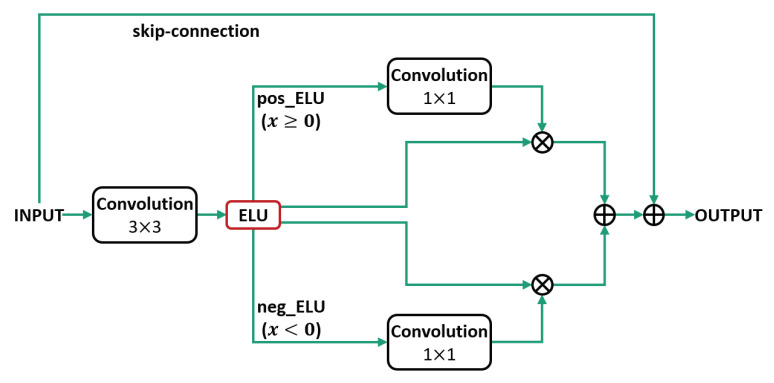
Neg-Region Attention module architecture.

We propose NRA to suppress the non-object area of the positive region and emphasize the object area using the negative region of the ELU. The proposed method was separately emphasized in the negative and positive regions after applying the ELU activation function. Figure 4 shows the proposed NRA structure.

The proposed NRA method is configured as follows after applying ELU, the process of separating the negative and positive areas results in an ELU feature map and an element-wise product spatial attention course using a 1 × 1 convolution in the separated area and an element of the emphasized feature map, which consists of a joint process using an element-wise sum. In Figure 4, neg_ELU represents the negative region of the ELU activation function and pos_ELU represents the positive region.

**Figure 5:**
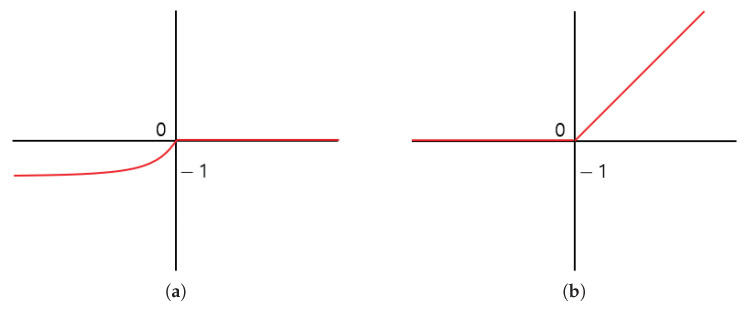
The separated ELU activation function graph. (**a**) is the negative region and (**b**) is the positive region.

Equation (2) is the process of separating into a negative region (neg_ELU) and a positive region (pos_ELU), which are respectively shown in (3) and (4), respectively. Figure 5 is a graph showing the separation in the ELU.

(3) $$ELU_{n}\left(x\right)=\left\{\begin{array}{cc} 0, & \mathrm{if}\;x\geq 0\\ a(e^{x}-1), & \mathrm{if}\;x<0 \end{array}\right.,a=1$$

(4) $$ELU_{p}\left(x\right)=\left\{\begin{array}{cc} x, & \mathrm{if}\;x\geq 0\\ 0, & \mathrm{if}\;x<0. \end{array}\right.$$

Using the ELU activation function, the feature map was separated into a negative area and a positive area. In Equation (3), $ELU_{n}\left(x\right)$ is the negative region of the ELU when the input is *x*, and the positive region is treated as 0. Because *a* is set to 1 and converges to −1, it can be confirmed that the texture information with a small amount of data change is unified and displayed. In Equation (4), $ELU_{p}\left(x\right)$ is a positive region of the ELU when the input is *x* and is processed in the same way as the ReLU function. The proposed method separates the positive and negative regions to take advantage of the properties of the ELU.

**Figure 6:**
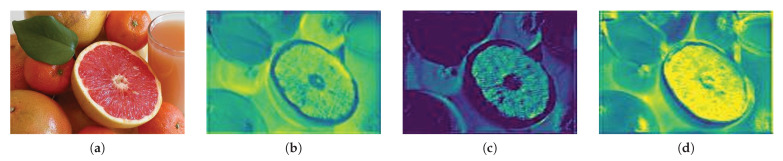
The ELU activation function and separated results. (**a**) is the input image and (**b**) is the ELU activation function results, (**c**) is the positive region, (**d**) is the negative region.

Figure 6 shows the module of Figure 4, which has a feature map separated into positive and negative regions, as shown in Figure 5. Figure 6c is a feature map showing the separation in the ELU. This map is different from that when the ReLU activation function is applied in the separation of the ELU feature map (b) into a positive region, so the normalization range is different and ReLU shows different results. In the positive region, various textures and boundary information are extracted according to the amount of data change in the image. These features are affected by color and brightness. The texture information of the salient object of lighting is extracted from the features of the non-object area, and the shadow features of the non-object area are extracted in the same way as the salient object. Figure 6d shows the results of dividing the saliency map into negative and positive regions, where a converges to −1.

#### 3.1.1. Extraction of Attention Region from Each Region

Because the SOD needs to detect the area of the salient object, not only the boundary information but also the texture information of the object to be detected is important. Various texture information can be obtained by outputting features through an ELU function with a wide range of feature expressions. However, environmental conditions, such as light reflection, affect color and brightness, and texture information is extracted based on such conditions, resulting in a loss area. Although the loss can be minimized by unifying the various texture information through an emphasis technique, a region with texture information similar to that of a salient object is emphasized, resulting in false detection. Therefore, the proposed method does not use the ELU function as it is and proceeds with the enhancement technique by separating the saliency map into a positive region representing various texture information and a negative region containing mainly unified texture information and boundary information with a large amount of change.

Areas of objects and backgrounds in the image are separated based on the boundary information. Spatial attention can be used for positive areas that have various textures, and boundary information can be used to unify the information of various textures and emphasize the salient object area. The feature separated into the negative region is different from that in the positive region, such that the feature converges to −1 and outputs unified texture information and boundary information with a large amount of data change. If these features are utilized without emphasis, then the area of the salient object can be suppressed as a non-object area. Spatial attention can be used to emphasize only the salient object area based on the boundary information to suppress the non-object area emphasized in the positive area.

The proposed method performs spatial attention in the positive and negative regions using Equations (5) and (6), respectively, and is a transformation of spatial attention. In the case of using the average and maximum pooling, a representative value is outputted in the separate positive region, resulting in a loss of various texture information. Because the sigmoid is normalized to a value between 0 and 1, the amount of data change is altered, so the boundary information is lost. Therefore, in the proposed method, spatial attention through a convolution and element-wise product is used without pooling and sigmoid.

(5) $$A_{p}\left(x\right)=f_{conv}^{1\times 1}\left(ELU_{p}\left(f_{conv}^{3\times 3}\left(x\right)\right)\right)⨂ELU\left(f_{conv}^{3\times 3}\left(x\right)\right)$$

(6) $$A_{n}\left(x\right)=f_{conv}^{1\times 1}\left(ELU_{n}\left(f_{conv}^{3\times 3}\left(x\right)\right)\right)⨂ELU\left(f_{conv}^{3\times 3}\left(x\right)\right).$$

**Figure 7:**
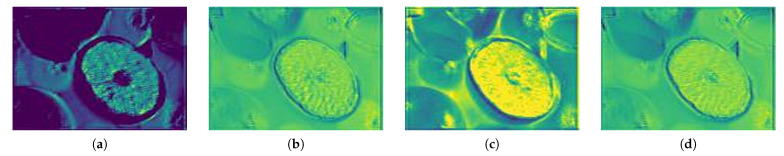
Results of applying attention in each area. (**a**) is the positive region and (**b**) is the positive attention results, (**c**) is the negative region, (**d**) is the negative attention results.

In Equations (5) and (6), $A_{p}\left(x\right)$ and $A_{n}\left(x\right)$ represent the positive and negative regions of the input *x*; $f_{conv}^{1\times 1}$ and $f_{conv}^{3\times 3}$ are the 1×1 and 3×3 convolutions, respectively; the ⨂ is an element-wise product; $ELU_{p}$ and $ELU_{n}$ are the positive and negative regions separated from the ELU in Equations (3) and (4), respectively; and *a* was set to 1 in the ELU. The results of such a modified spatial attention equation are shown in Figure 7.

Figure 7 shows a feature map of each region separated from the ELU and a feature map emphasizing the positive and negative regions. Spatial attention was performed on the salient object to emphasize the detailed information from Figure 7a. Negative areas also emphasized the contour and texture information via spatial attention. Unlike the result of applying the ELU activation function, the area of the salient object was emphasized. The emphasis of the positive area emphasizes the texture information of the entire image, and the negative area suppresses the non-object area and emphasizes the contour and texture information.

#### 3.1.2. Combination of Attention Positive and Negative Region

The texture information of the salient object was emphasized based on the boundary information between the salient object and the background, and the positive area where the shadow area was emphasized and the negative area where the non-object area was suppressed were combined with the element-wise sum. As a result of emphasizing the negative region through the combination, the shadow region, which is a non-object region, is suppressed, and the feature that the region of the salient object is emphasized through the emphasis on the positive and negative regions is obtained. Equation (7) shows the combination of the emphasized feature maps.

(7) $$A_{element}\left(x\right)=A_{p}\left(x\right)+A_{n}\left(x\right).$$

The combination of the feature maps emphasized for input *x* is represented by $A_{element}\left(x\right)$. $A_{p}$ represents a feature map with emphasized positive areas, and $A_{n}$ represents a feature map with emphasized negative areas.

**Figure 8:**
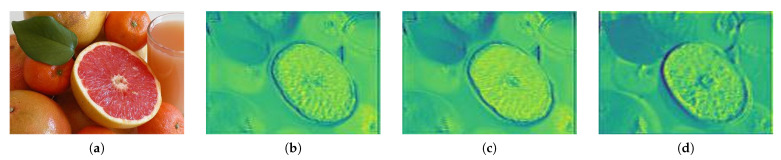
Sum of the elements of the attention result for each region. (**a**) is hte input image and (**b**) is the positive attention result, (**c**) is the negative attention result, (**d**) is the element-wise sum result.

Figure 8 shows the result of a combination of feature maps and shows an emphasis on each area. Bases on the spatial attention results of the positive region, which contains various detailed information, we have element-wise-summed the positive and negative feature maps emphasized to suppress the shadow features that are non-object regions. By combining the results of emphasizing the unified texture information in (c) and the result of emphasizing various texture information in (b), the non-object area is suppressed, as shown in Figure 8d. The boundary and texture information of the salient objects is also emphasized.

In the deep learning process, when the distance between the input and output increases as shown above, the slope value is saturated with a large or small value in the backpropagation process in which the weight is transmitted between layers when learning the network, resulting in an ineffective learning, a slope that slows learning, and loss problem. To prevent these problems, a structure that learns the difference between the input value and output value was constructed by applying skip connection after combining the highlighted feature maps.

**Figure 9:**
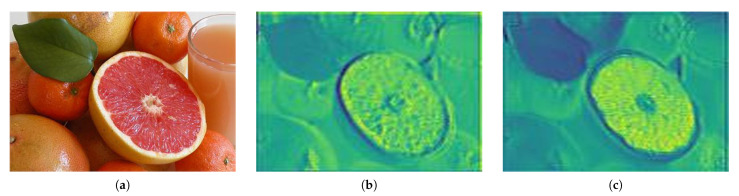
The skip-connection after element-wise sum. (**a**) is the input image and (**b**) is the element-wise sum result, (**c**) is the skip-connection result.

Figure 9 compares the results of the highlighted feature map combination with the results of applying skip connection to the combined feature map. The problems of weights being propagated directly from the output to the input and the slope disappearing in deep structures when learning the network through a structure that applies skip connection are avoided. The final NRA result is shown in Figure 9c, in which the non-object area is suppressed and the detailed information of the object is emphasized. Equation (8) is the formula for NRA.

(8) $$A_{NA}\left(x\right)=A_{element}\left(x\right)+x.$$

The result of NRA is represented by $A_{NA}\left(x\right)$ on the input *x*, and $A_{element}\left(x\right)$ and the input are element-wise-summed to utilize skip connection. $A_{element}\left(x\right)$ is a combination of the positive and negative feature maps emphasized in Equation (7).

**Figure 10:**
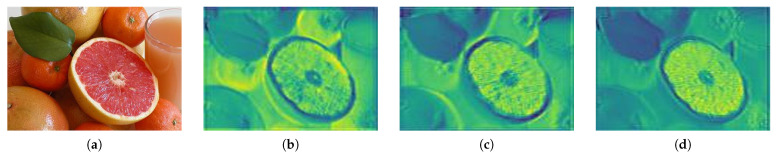
The Neg-Region Attention module results. (**a**) is the input image and (**b**) is the ELU activation function result, (**c**) is the attention result using the ELU activation function, (**d**) is the Neg-Region Attention module result.

Figure 10 is a comparison of the feature map to which the ELU activation function is applied when performing the convolution operation on the input image and the result of applying the proposed NRA to the result emphasized by these feature maps. When spatial attention is applied to the feature map extracted using the ELU activation function, the texture information of the salient object is extracted in the non-object area by lighting, as shown in Figure 10c. The proposed NRA method is separated into positive and negative regions by the ELU activation function, and then spatial attention is applied to each region to suppress non-object regions and emphasize the detailed information of salient objects.

### 3.2. Decoder for Extending Extracted Features

Because the decoding process is a stage where feature extraction and compressed features are expanded to the size of the input image and restored at the encoder stage, feature loss occurs during the expansion process. In this process, information on the correlation of surrounding pixels is lost. The proposed method uses concatenation to utilize the features of each stage extracted from the encoder at each stage of the decoder to prevent feature loss.

Unlike skip connection, which adds a feature map, concatenation simply follows. The number of feature maps is increasing, following the feature maps of the same size. Equation (9) is the formula for concatenation:

(9) $$Concat\left(w,h\right)=[A_{NA}{\left(x\right)}_{w\times h};D{\left(y\right)}_{w\times h}].$$

$A_{NA}\left(x\right)$ is the result of performing NRA on the input *x*, and w×h indicates the size of the resulting feature map. D(y) is the result of the inverse convolution of the input *y* with the decoder, and w×h indicates the size of the result feature map. Concat(w,h) is the result of the concatenation of a feature map of the same size as w×h.

### 3.3. Residual Block in the Process of Concatenation Operation

**Figure 11:**
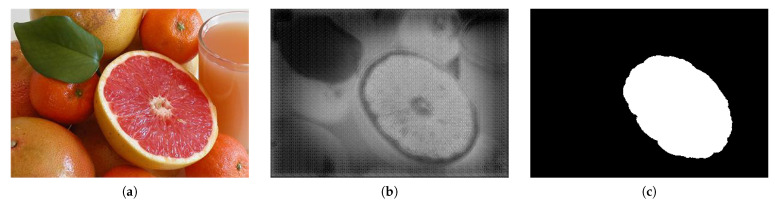
Prediction result of the model using only the attention module. (**a**) is the input image and (**b**) is using only the attention module, (**c**) is groundtruth.

When the NRA result of the encoder step is directly concatenated to the decoder, only the salient region is expressed because the highlighted feature is not refined. A residual block was used to generate a saliency map close to the ground truth through the feature refinement process of this highlighted region. Figure 11 shows the results of the concatenation of the NRA without feature refinement.

The results show only the emphasized regions where the features have not been purified. The features in the residual block were reconstructed to improve the quality of the saliency maps and generate them closer to the ground truth.

The existing residual block consists of two (3×3) convolutions and two ReLU activation functions. Such a structure does not take advantage of the negative region features using the ReLU activation function. Because the proposed method also utilizes features in the negative region, we used the ELU activation function to prevent the loss of features highlighted by the residual block. The information transmitted by skip connection in the proposed method emphasizes the salient area. When the ELU activation function was applied after receiving the emphasized feature information, the features in the emphasized negative region were normalized and feature loss occurred. Therefore, unlike the conventional method, the result was outputted without using the activation function after skip connection. Equation (10) is an equation of the residual block by the proposed method.

(10) $$f_{res}\left(x\right)=f_{conv}^{3\times 3}\left(ELU\left(f_{conv}^{3\times 3}\left(x\right)\right)\right)+x.$$

**Figure 12:**
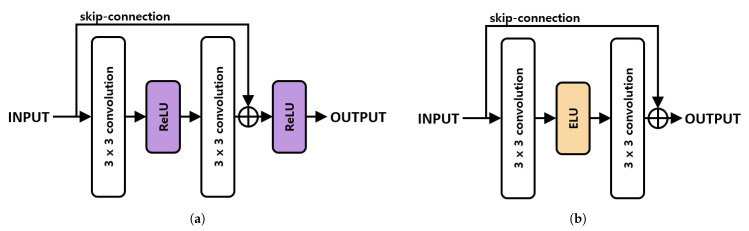
Composition of residual blocks. (**a**) is existing residual block and (**b**) is residual block.

$f_{res}\left(x\right)$ is the result of the residual block in the proposed way of the input *x*, and $f_{conv}^{3\times 3}$ is the 3×3 convolution. After extracting the features in the 3×3 convolution as the input, the features were enabled as an ELU function and the features were extracted again via a 3×3 convolution. Then, the input was added to the element-wise sum, and the skip-connection structure was used. Figure 12 shows the structure of the residual block.

**Figure 13:**
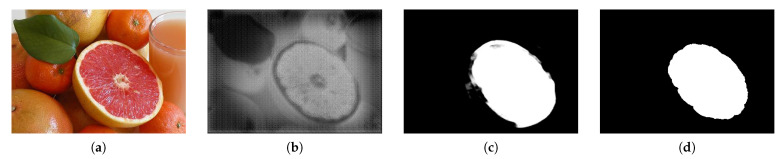
Results of using the proposed residual block. (**a**) is the input image and (**b**) is using only the attention module, (**c**) is using the proposed residual block. (**d**) is ground truth.

The features of the structure of these proposed residual blocks were purified. This method also avoids the problem of slope disappearance in the skip connection, reduces loss, and generates a saliency map close to the ground truth. Figure 13 shows the final SOD results.

## 4. Experimental Environment

### 4.1. Environment and Dataset

In this paper, MSRA10K (10,000) Salient Object Database was used as the training dataset, and ECSSD (1000) was used as the validation dataset. The MSRA10K dataset was trained with a total of 80,000 images using rotation (0${}^{\circ }$, 90${}^{\circ }$, 180${}^{\circ }$, 270${}^{\circ }$) and flipping. During the learning process, the verification dataset was used to compare the degree of convergence between the other datasets and to confirm the overfitting phenomenon in which the accuracy of only the training dataset increases. Experimental datasets were compared and analyzed using ECSSD, HKU-IS (4447), and DUT-OMRON (5182). Adaptive Moment Estimation (Adam) optimizer [14] was used as the optimization function. The initial learning rate was set to 0.0001, the batch size was set to 48, and the epoch was set to 80. GPU was trained and experimented using NVIDIA GeForce RTX 3090 24 GB. The learning rate was set through a number of experiments, and if the initial learning rate exceeds 0.0001, the learning convergence speed is fast and learning is not performed. It was adjusted through the learning rate scheduler according to the learning convergence speed. The size of the input image was set to 224 × 224, which is the most used for comparison with existing methods.

### 4.2. Loss Function

The proposed method uses the L2 loss function. It is used when there is only one type of object to be detected, such as SOD, or when only correct and incorrect answers are identified. When there are various types of objects to be detected, such as object recognition, loss is calculated for each type of object using cross-entropy. The L2 loss function calculates the error by comparing the saliency map predicted with the mean squared error (MSE) and the squared error of the ground truth. When calculating the error, there are outliers in which the value rapidly changes. MSE is greatly affected by these outliers, and the weights are adjusted accordingly. Equation (11) is the equation of the L2 loss function.

(11) $$f_{Loss}\left(x\right)=\sum_{i=1}^{n}{\left(y_{i}-\hat{y_{i}}\right)}^{2}.$$

*y* represents the ground truth, and $\hat{y}$ represents the saliency map predicted by the proposed method. The result of summing the difference between the ground truth and the predicted saliency map is $f_{Loss}$, and reducing this value entails the weight adjustment of the learning process.

### 4.3. Evaluation Index

To compare and analyze the experimental results, the mean absolute error (MAE) [15], precision, recall, and F-measure [16] were used as evaluation indicators. Equation (12) is an expression of the evaluation index MAE.

(12) $$MAE=\frac{1}{W\times H}\sum_{x=1}^{W}\sum_{y=1}^{H}\left|S(x,y)-G(x,y)\right|.$$

S(x,y) represents the predicted saliency map, and G(x,y) represents the ground truth. W×H represents the size of the image. MAE is an error rate that represents the absolute error value between the ground truth and the predicted result, so the lower the value, the better the performance. Equation (13) is the expression of the precision and recall, and Equation (14) is the F-measure.

(13) $$precision=\frac{TP}{TP+FP},recall=\frac{TP}{TP+FN}$$

(14) $$F_{\beta }=\left(1+\beta^{2}\right)\frac{precision\times recall}{(\beta^{2}\times precision)+recall},\beta^{2}=0.3.$$

The precision, recall and F-measure are values that indicate accuracy, and a value higher than 0 indicates better performance. Precision and recall are calculated based on whether the ground truth and the pixel value at the same location in the saliency map are the same.

S-measure (Structure-measure) [17] simultaneously evaluates object-aware structural similarity and region-aware between a predicted saliency map and a ground truth. S-measure is Equation (15).

(15) $$S=\alpha \times S_{o}+\left(1-\alpha \right)\times S_{r},$$

where $S_{o}$ is the object-aware structural similarity measure and $S_{r}$ is the region-aware structural similarity measure. S-measure is a combination of two evaluations, and α=0.5 was used.

E-measure(Enhanced-alignment measure) [18] combines the image-level mean value and local pixel values into one. Jointly capture local pixel matching information and image level statistics. E-measure is defined by Equation (16).

(16) $$Q_{FM}=\frac{1}{W\times H}\sum_{x=1}^{W}\sum_{y=1}^{H}\phi_{FM}\left(x,y\right),$$

where *h* and *w* are height and width of the map.

## 5. Experimental Results

### 5.1. Learning Convergence Experiment

**Figure 14:**
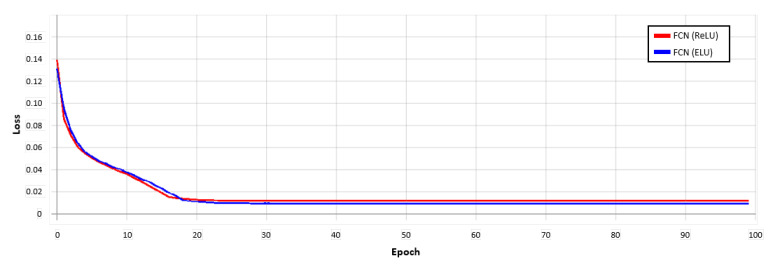
Loss convergence graph for each activation function in the FCN model. Red used the ReLU function, and blue used the ELU function.

Figure 14 is a comparative analysis graph of the loss convergence and precision convergence in the learning process of the conventional FCN method using the ReLU activation function and the FCN method using the ELU activation function. The loss converged faster in the learning process of the FCN (ELU) method using the ELU activation function than the conventional FCN (ReLU). The findings confirmed that using ELU instead of ReLU resulted in a faster convergence.

### 5.2. Training MSRA10K Dataset

**Figure 15:**
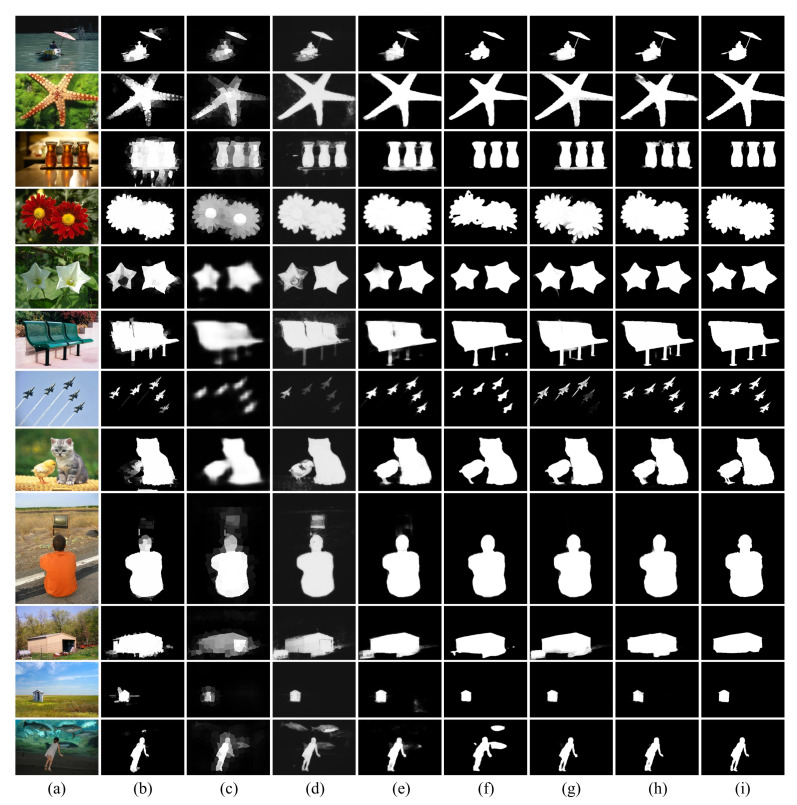
Comparison of experimental results of the proposed method and other deep learning methods. (**a**) Input image, (**b**) ELD, (**c**) DS, (**d**) DCL, (**e**) Amulet, (**f**) DGRL, (**g**) AFNet, (**h**) NRA-Net (proposed method), (**i**) Groundtruth.

Figure 15 is a comparison image of the proposed method and other existing methods and experimental results. The experimental results were compared with ECSSD (4 images), HKU-IS (4 images), and DUT-OMRON (4 images) as examples, and the comparison methods were ELD (Encoded Low level Distance map) [19], DS (Deep Saliency) [20], DCL (Deep Contrast Learning) [21], Amulet [22], DGRL (Detect Globally Refine Locally) [23] and AFNet [24], which are all deep learning-based methods. All of these methods use a backbone network, whereas the proposed method was trained without a backbone network. Other detectors use the backbone network for the encoding process, which is the feature extraction step, so various features can be easily extracted. However, the proposed method improves the performance of the feature extraction step by applying the NRA without a backbone to the encoding step and minimizes the loss of texture and contour information. ELD and DS were greatly affected by color and brightness, and detected the surroundings of the target object. DCL mainly detected a single object and detects other objects together. It was vulnerable to multi-object detection and showed a result that is sensitive to contour information. The Amulet detects the area of the target object, but if the input image is complex, the surroundings are also detected. In some of the result images, a background area other than the surrounding area was also detected. DGRL showed a clear detection results, but loss occurred in the detailed part and the surrounding area was detected together. AFNet showed the best performance compared to the previous methods and the area and contour of the object were preserved. If there were multiple small objects, some detection fails, and if the background was complex, the surroundings were detected together. The proposed method showed excellent detection performance for small objects and detected large and multiple objects well. As with the existing methods, when the background is complex, the surroundings were detected together, but false detection was reduced. When compared to existing methods by learning without a backbone network, it showed excellent performance, and the performance of the attention module using ELU was also proven.

Table 1 and Table 2 are comparison tables for the evaluation of the proposed method and other deep learning-based algorithms. The number of parameters of the proposed method is less than the average and shows excellent performance without using the backbone network. The MAE, mean F-measure ($\beta^{2}=0.3$), S-measure and E-measure were measured for the datasets ECSSD, HKU-IS, and DUT-OMRON. The best performance numbers are expressed in red, the second is blue, and the third is green. The proposed method showed superior performance in the MAE, mean F-measure, S-measure and E-measure compared to the conventional methods using the backbone network.

**Figure 16:**
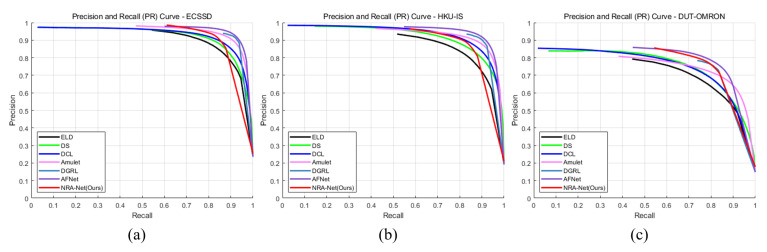
Comparison with other methods of precision and recall curve in each dataset. (**a**) PR curve of ECSSD dataset and (**b**) HKU-IS dataset, (**c**) DUT-OMRON dataset.

**Figure 17:**
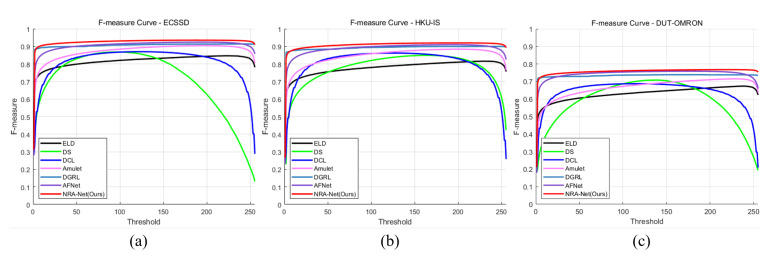
Comparison with other methods of F-measure curve in each dataset. (**a**) F-measure curve of ECSSD dataset and (**b**) HKU-IS dataset, (**c**) DUT-OMRON dataset.

Figure 16 and Figure 17 are the comparison diagrams of the precision and recall curves and F-measure curves of the proposed method and other deep learning algorithms. The proposed method shows excellent performance in both indicators. In this curve, the minimum recall value can be used as an indicator of robustness, where the higher the precision value of the minimum recall value, the more accurate the salient object prediction, which means that the background and foreground are well separated.

**Figure 18:**
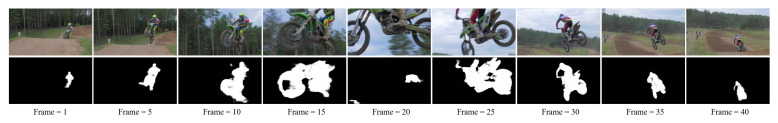
Salient object detection results for motocross-jump video of MIT300 dataset. (**top**) Input video frame and (**bottom**) results of saliency map.

Figure 18 shows the result of detection of protruding objects for the motocross-jump video dataset. When performing gaze tracking, gaze detection may fail if a dramatic scene change occurs as in the video above. When a dramatic scene change occurs, there are factors such as the position of the object or the rotation of the camera. The detection of salient objects compensates for this problem and predicts objects in which human gaze is concentrated even with scene changes. This prediction result can supplement information on which object is mainly focused on gaze detection.

## 6. Conclusions

In this paper, we propose a deep learning-based method to detect salient objects in images in various environments. Existing deep learning-based methods proceed with an autoencoder structure, and feature loss occurs in the encoding process for extracting and compressing features and the decoding process for expanding and restoring the extracted features. Due to this feature loss, a background other than an object is detected, or an object with complex internal information fails to be detected. Most of the existing methods require a backbone network, and improve the network based on the backbone. However, feature extraction is limited, and it is difficult to extract specialized features for any object. The efficiency of the proposed method to reduce the feature loss in the autoencoder structure was studied. After separating the positive and negative regions through the NRA proposed in the encoding process of the autoencoder structure, the enhancement technique was performed. Positive numbers represent various textures and boundary information, and negative numbers mainly represent boundary information with a large amount of change in data. To utilize this characteristic information, spatial attention technique was performed in each domain. The proposed method prevents feature loss and creates a final saliency map by reconstructing features with a modified residual block. Existing deep learning methods extract features using a backbone network, but the proposed method achieves an excellent performance by extracting features using the attention technique without a backbone network.

## Figures and Tables

**Table 1:** The experimental results of the ECSSD dataset.

Method	Number of Parameters	ECSSD Dataset			
		MAE	F-Measure	S-Measure	E-Measure
ELD	28.37 M	0.0796	0.8102	0.838	0.881
DS	134.27 M	0.1216	0.8255	0.820	0.874
DCL	66.25 M	0.1495	0.8293	0.863	0.885
Amulet	33.16 M	0.0588	0.8684	0.893	0.901
DGRL	126.35 M	0.0419	0.9063	0.903	0.917
AFNet	21.08 M	0.0422	0.9085	0.913	0.918
NRA-Net	56.42 M	0.0489	0.9126	0.898	0.907

**Table 2:** The experimental results of HKU-IS and DUT-OMRON datasets.

Method	HKU-IS Dataset				DUT-OMRON Dataset			
	MAE	F-Measure	S-Measure	E-Measure	MAE	F-Measure	S-Measure	E-Measure
ELD	0.0741	0.7694	0.820	0.880	0.0923	0.6110	0.750	0.775
DS	0.0780	0.7851	0.852	0.889	0.1204	0.6031	0.750	0.761
DCL	0.1359	0.8533	0.860	0.913	0.0971	0.6837	0.764	0.801
Amulet	0.0521	0.8542	0.883	0.910	0.0977	0.6474	0.780	0.778
DGRL	0.0363	0.8882	0.894	0.943	0.0618	0.7332	0.806	0.848
AFNet	0.0364	0.8904	0.905	0.942	0.0574	0.7382	0.826	0.853
NRA-Net	0.0428	0.8924	0.894	0.919	0.0706	0.7449	0.811	0.836

## Data Availability

Code and dataset will be made available on request to the first author’s email with appropriate justification. The public site for each dataset is as follows. MSRA10K: https://mmcheng.net/msra10k/; ECSSD: http://www.cse.cuhk.edu.hk/leojia/projects/hsaliency/dataset.html; DUT-OMRON: http://saliencydetection.net/dut-omron/; HKU-IS: https://sites.google.com/site/ligb86/hkuis.
